# Genome-Wide Analyses Identify Filamin-A As a Novel Downstream Target for Insulin and IGF1 Action

**DOI:** 10.3389/fendo.2018.00105

**Published:** 2018-03-20

**Authors:** Daniel Aizen, Metsada Pasmanik-Chor, Rive Sarfstein, Zvi Laron, Ilan Bruchim, Haim Werner

**Affiliations:** ^1^Department of Human Molecular Genetics and Biochemistry, Sackler School of Medicine, Tel Aviv University, Tel Aviv, Israel; ^2^Bioinformatics Unit, George Wise Faculty of Life Sciences, Tel Aviv University, Tel Aviv, Israel; ^3^Endocrine and Diabetes Research Unit, Schneider Children’s Medical Center, Petah Tikva, Israel; ^4^Gynecological Oncology Division, Hillel Yaffe Medical Center, Technion – Israel Institute of Technology, Hadera, Israel; ^5^Yoran Institute for Human Genome Research, Tel Aviv University, Tel Aviv, Israel

**Keywords:** insulin-like growth factor-1, insulin analogues, endometrial cancer, microarray analysis, filamin-A

## Abstract

Insulin analogs were developed to improve diabetes therapy. However, certain modifications introduced into the insulin molecule were shown to enhance their affinity to the insulin-like growth factor-1 receptor (IGF1R). Most tumors, including endometrial cancers, express high levels of IGF1R. The present study was aimed at identifying the entire set of genes that are differentially activated by insulin glargine or detemir, in comparison to insulin and IGF1, in Type 1 and Type 2 endometrial cancer cell lines (ECC-1 and USPC-1, respectively). Global gene expression analyses demonstrated a ligand-dependent upregulated expression of filamin-A (FLNA), a gene that encodes an actin filament cross-linking protein, in both endometrial cancer cell types. Silencing experiments linked to migration assays confirmed the role of FLNA in cell growth and motility. Our data suggest that the activation of distinct sets of genes by glargine may lead to stimulation of specific pathways or, alternatively, may provide additive effects, different from those classically induced by insulin. Given that metastases are probably the main factor contributing to tumor invasiveness, the identification of FLNA as a downstream target for insulin-like hormones may be of translational relevance in oncology. Clinical studies in endometrial cancer may add further relevant information regarding the possible differential actions of insulin analogs with respect to native insulin.

## Introduction

Insulin analogs were developed in order to provide a better glycemic control to diabetes patients. Analogs are generated by modifying the amino acid sequence of the insulin molecule to either prolong or shorten their action time (1–3). These modifications usually lead to improved pharmacokinetic and/or pharmacodynamic properties. Insulin analogs are classified into two main categories: short-acting and long-acting analogs, according to their action kinetics and absorption rates. However, certain structural changes introduced into the insulin molecule were shown to alter the affinities of analogs for the insulin (INSR) and insulin-like growth factor-1 receptor (IGF1R) (4). Altered binding kinetics may lead to a number of non-classical biological activities often associated with insulin analogs.

Most clinical and experimental data support the concept that INSR activation (primarily by insulin) leads to metabolic types of action. On the other hand, IGF1R activation (primarily by IGF1 or IGF2) is mainly linked to growth and differentiation (5, 6). Nonetheless, there is a large degree of cross talk within this growth factor family. These complex interactions seem to be responsible for compensatory and overlapping activities usually observed in this endocrine axis. The IGF1R, which is structurally related to the INSR, is expressed in many types of cancer and is regarded as an important player in the establishment of a malignant phenotype. Given that some insulin analogs display an enhanced affinity to IGF1R, reports have shown that various analogs exhibit increased mitogenic activities in comparison to native insulin (7–10). The clinical implications of these findings, however, are still a matter of debate (4, 11, 12).

Endometrial cancer is one of the most common types of gynecological cancer (13). Endometrial cancers are divided into Type I and Type II tumors (14). Type I tumors account for ~80% of cases, are usually well-differentiated, and have a relatively good prognosis. Type II tumors, on the other hand, are more aggressive, occur in older women, and have a worse prognosis. The incidence of endometrial cancer significantly increased in recent years, apparently as a result of the growing obesity epidemics (15). Based on epidemiological and clinical correlations, we postulated that insulin analogs might elicit non-classical mitogenic and signaling activities in endometrial cancer. In a recent study, we analyzed the actions of short-acting insulin lispro (LysB28, ProB29 human insulin) and aspart (AspB28 human insulin), and long-acting insulin glargine (GlyA21, ArgB31, ArgB32 human insulin), and detemir [LysB29 (N-tetradecanoyl) des (B30) human insulin] in endometrial cancer cells (16). We showed that glargine exhibits a strong, IGF1-like, mitogenic effect whereas detemir and short-acting analogs lispro and aspart display reduced proliferative potentials. In addition, analogs activated the INSR and IGF1R pathways in cell-type specific fashions.

The present study was designed to identify the entire set of genes that are differentially activated by glargine or detemir, in comparison to native insulin and IGF1, in both Type I and II endometrial cancer cell lines. Global gene expression analyses allowed us to identify the molecular events that are differentially induced by analogs, as well as genes that are commonly activated by all insulin-like hormones. The filamin-A (*FLNA*) gene encodes an actin filament cross-linking protein that interacts with binding proteins involved in cell motility. Microarray gene expression analyses showed a significant ligand-dependent upregulated expression of FLNA in ECC-1 and USPC-1 endometrial cancer cells. Silencing experiments linked to migration assays confirmed the role of FLNA in cell growth and motility. Hence, our data identify FLNA as a novel downstream target candidate for insulin-like hormones.

## Materials and Methods

### Cell Cultures

Gene expression analyses were conducted in the Type I endometrial cancer cell line ECC-1 and Type II uterine serous papillary carcinoma cell line USPC-1. ECC-1 cells were grown in DMEM and USPC-1 cells in RPM-1640 media (Biological Industries, Beit Haemek, Israel). Media were supplemented with 10% fetal bovine serum, 2 mM glutamine, 50 µg/ml gentamicin sulfate, and 5.6 mg/l amphotericin B. The ECC-1 cell line was obtained from Dr. Yoav Sharoni (Ben Gurion University, Beer-Sheba, Israel) and the USPC-1 cell line was provided by Dr. Alessandro Santin (Yale University School of Medicine, New Haven, CT, USA). Cells were treated with the following hormones: regular insulin (Humulin R^®^, Lilly Pharma, Germany), insulin glargine (Lantus^®^, Sanofi Aventis, Germany), insulin detemir (Levemir^®^, Novo Nordisk, Denmark) and IGF1 (PeproTech Ltd., Rocky Hill, NJ, USA).

### RNA Extraction and cDNA Synthesis

ECC-1 and USPC-1 cells were seeded in 10-cm Petri dishes and grown until confluence. Cells were serum-starved for 24 h and treated with insulin, IGF1, glargine, or detemir, at a dose of 8.6 nM, for 8 h. After incubation, cells were washed with PBS, and total RNA was extracted using the TRIzol^®^ Reagent (Life Technologies, Carlsbad, CA, USA). RNA was quantitated by measuring absorption at 260/280 and 260/230 nm using a NanoDrop 2000 spectrophotometer (Thermo Fisher Scientific, Wilmington, DE, USA). Extracted RNA was converted to single-stranded cDNA using a High Capacity cDNA Reverse Transcription Kit (Applied Biosystems, Carlsbad, CA, USA).

### Genomic Analyses

Affymetrix^®^ Human Gene 2.1 ST Array Strips (Cat. #902114, Affymetrix, Santa Clara, CA, USA) were used for gene expression analysis. These microarrays offer whole-transcript coverage of 30,654 well established annotations of total RefSeq transcripts with 764,885 distinct probes. The microarray data was submitted to GEO and assigned the accession GSE109022.

### Bioinformatics Analyses

Data analysis was performed on CEL files using Partek Genomics Suite v 6.6 (Partek, St. Louis, MO, USA). Data were normalized and summarized with the robust multi-average method followed by one-way analysis of variance. Differentially expressed genes were selected by using *p*-value (FDR corrected) <0.05 and fold change (FC) of at least 1.5. Hierarchical clustering analysis was done by Partek Genomics Suite software with Pearson’s dissimilarity correlation and average linkage methods.

### Real-Time Quantitative Polymerase Chain Reactions (RT-qPCR)

Real-time quantitative PCR of genes of interest was performed in a StepOne™ Real-Time PCR System (Applied Biosystems™, Life Technologies Ltd., UK) by using the FastStart Universal SYBR Green Master (Rox) (Roche, Mannheim, Germany). Primer sequences for RT-qPCR are shown in Table 1. The expression measurement of the designated genes was analyzed using the StepOne™ Real-Time PCR software. Primers were normalized by specific cDNA standard curves obtained from known amounts of cDNA. The comparative Ct method was used for presenting quantitative data. RT-qPCR experiments were performed at least three times, in triplicates for each gene product examined. A SD for each relative gene expression value was calculated as a measure of data variation.

**Table 1:** Primer sequences for validation of microarray results by real-time quantitative polymerase chain reactions.

Primers	Sequences (5′–3′)	Annealing (°C)	Product length (bp)
Block of proliferation (BOP1)-F	GGAGGACAGCTCTGATGAGG	64.0	123
BOP1-R	CGCAGGGGCTTGTAGATG	64.5	
CCNE1-F	ACAGCTTGGATTTGCTGGAC	64.2	117
CCNE1-R	TCTGCTTCTTACCGCTCTGTG	64.5	
ERBB3-F	CCCTATGCAGGGCTACGA	63.8	130
ERBB3-R	TGTTCTCATCAATCATCCAACAC	63.7	
Filamin-A (FLNA)-F	CCCTTTCCTCTGGAAGCTGT	64.5	115
FLNA-R	CCTTGGTGTCGATGGTGAA	64.8	
HDAC5-F	CTGAATACCACACCCTGCTCT	63.1	109
HDAC5-R	CAAGGCAGCACAGCATACAT	63.9	
INSR-F	AACCCGACAACTGTCCAGAG	64.2	109
INSR-R	TCGTCCTTGAGCAGGTTGA	64.8	
Insulin receptor substrate-2 (IRS2)-F	TTCTTGTCCCACCACTTGAA	63.2	84
IRS2-R	CTGACATGTGACATCCTGGTG	64.3	
Ornithine decarboxylase (ODC1)-F	AAAACATGGGCGCTTACACT	63.3	112
ODC1-R	TGGAATTGCTGCATGAGTTG	64.9	
Actin-F	AGAGCTACGAGCTGCCTGAC	63.8	144
Actin-R	R-CGTGGATGCCACAGGACT	65.1	

### Western Blot Analyses

Cells were serum-starved overnight and then treated with insulin analogs, IGF1, or insulin at the indicated concentrations. After incubation, cells were harvested and lysed in a buffer containing protease inhibitors. Following centrifugation, the supernatants were collected and protein concentrations were determined. Samples were electrophoresed, followed by transfer to nitrocellulose membranes. Membranes were blocked with 5% skim milk for 1 h. The membranes were then incubated overnight with the indicated antibodies. After incubation, blots were washed and incubated with the appropriate horseradish peroxidase (HRP)-conjugated secondary antibody. The secondary antibodies were HRP-conjugated goat anti-rabbit IgG (1:50,000) and donkey anti-mouse IgG (1:25,000). Membranes were developed by using the SuperSignal West Pico^®^ Chemiluminescent Substrate (Pierce, Rockford, IL, USA) and analyzed using chemiluminescence imaging analyzer Fusion FX7 (Vilber Lourmat SAS, France).

### Knockdown of FLNA by siRNA Transfection

ECC-1 cells were seeded at a density of 1 × 10^6^/ml into 10-cm plates and incubated until 60% confluent. Filamin-A gene silencing was performed using filamin-A siRNA (Cat. #sc-35374, Santa Cruz Biotechnology), a pool of three target-specific 20–25 nt siRNAs designed for the inhibition of filamin-A expression in human cells. To obtain the best silencing efficiency, preliminary experiments were performed to determine the optimal concentration of siRNAs and the kinetics of silencing. Control siRNA-A (Cat. #sc-35374), containing a scrambled sequence of non-targeting 20–25 nt siRNA, was used as a negative control. Western blotting was performed to confirm the gene expression knockdown of FLNA in transfected cells.

### Cell Migration Assays

Cell migration assays were assessed by IncuCyte ZOOM™ CellPlayer 96-Well Kinetic (Essen Bioscience, Ann Arbor, MI, USA), which is designed for kinetic quantification of cell migration. Briefly, ECC-1 cells were seeded at a density of 3 × 10^4^ cells per well and incubated overnight in full media. Wound scratchs were made the following day using a 96-pin WoundMaker™ device and incubated in starvation media with or without increasing concentrations of ligands. Cell migration was monitored in real time and confluence was measured by the IncuCyte ZOOM™ software. Results were plotted as percentage wound confluence as a function of time.

### Statistical Analyses

The statistical significance of the differences between groups was assessed by Student’s *t*-test (two samples, equal variance) using SPSS Statistics version 19 software. Densitometry analysis was performed using FUSION-CAPT analysis software (Vilber Lourmat SAS, France). Signal intensities of phosphor-proteins were normalized to the corresponding protein signals. Data are presented as mean ± SE of three independent experiments. *p*-Values less than 0.05 were considered statistically significant.

## Results

### Microarray and Bioinformatics Analyses

In recent studies, we provided evidence that certain insulin analogs elicit differential activities in endometrial cancer cells in comparison to regular insulin or IGF1. In order to examine gene expression variation in endometrial cancer cells following treatment with insulin glargine, detemir, regular insulin, or IGF1 (in comparison to untreated control cells), the Affymetrix^®^ Human Gene 2.1 ST Array was employed. RNA was prepared from 15 ECC-1 samples (corresponding to three biological replicates for each treatment) and five USPC-1 samples (corresponding to one biological replicate per treatment), and subjected to gene expression array analysis. Differentially expressed genes following ligand treatment were selected by using *p*-value (FDR corrected) <0.05 and FC of at least 1.5.

**Figure 1:**
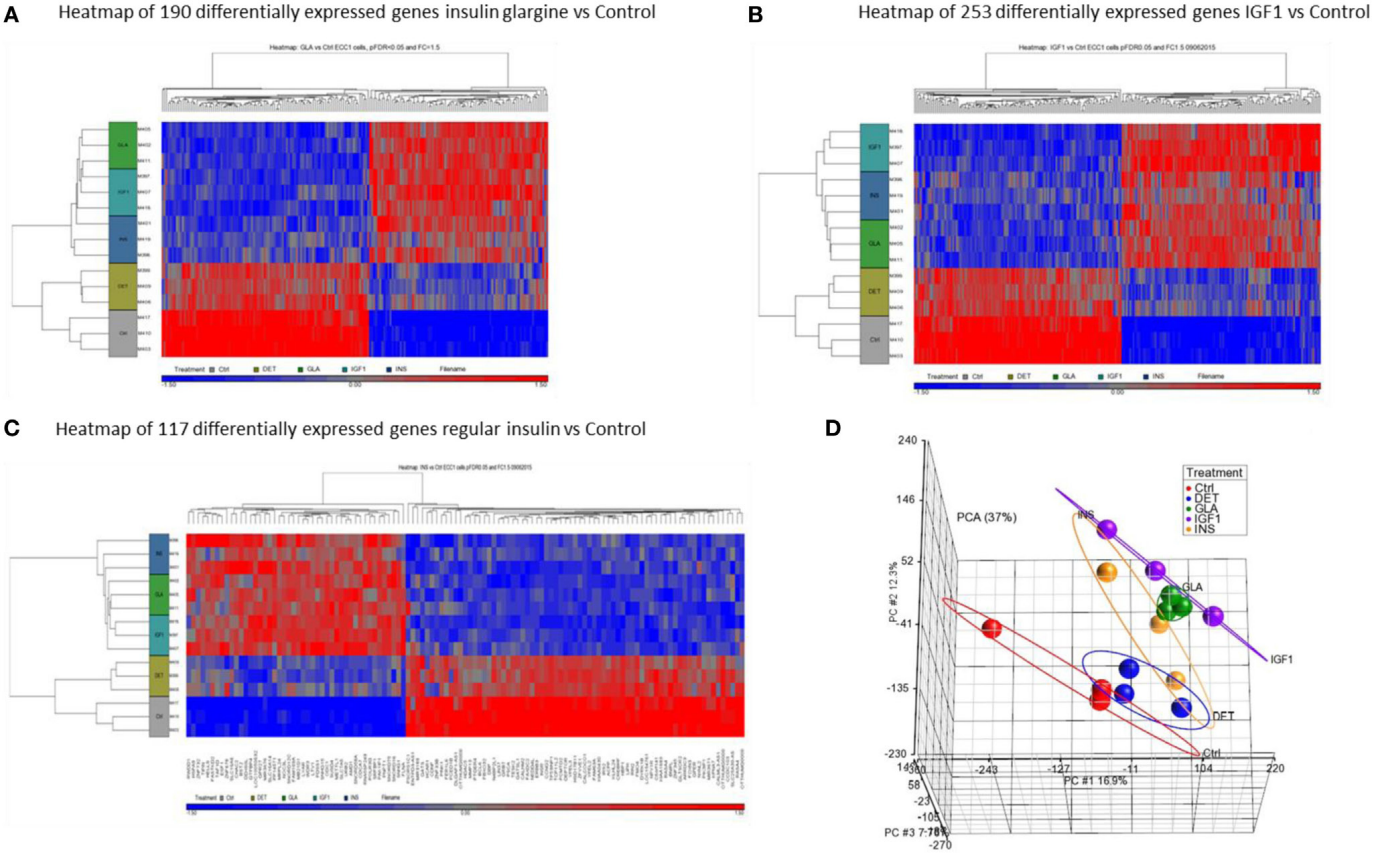
Hierarchical clustering map of differential gene expression in ECC-1 cells. Heatmaps of microarray results in the ECC-1 cell line after treatment with insulin glargine **(A)**, IGF1 **(B)**, and regular insulin **(C)**. The figures depict heatmaps of differentially expressed genes [FC > 1.5 or <−1.5 and *p*-value (FDR) < 0.05]. The scale below indicates the relative changes in gene expression represented by red (upregulated genes) and blue (downregulated genes) colors. **(D)** Principal component analysis (PCA) of all 15 arrays of ECC-1 cells (three biological replicates were performed for each treatment). Each data point represents RNA sample applied to microarray: red (untreated cells—Control, Ctrl), blue (Detemir, DET), green (Glargine, GLA), violet (IGF1), and orange (regular insulin, INS).

**Figure 2:**
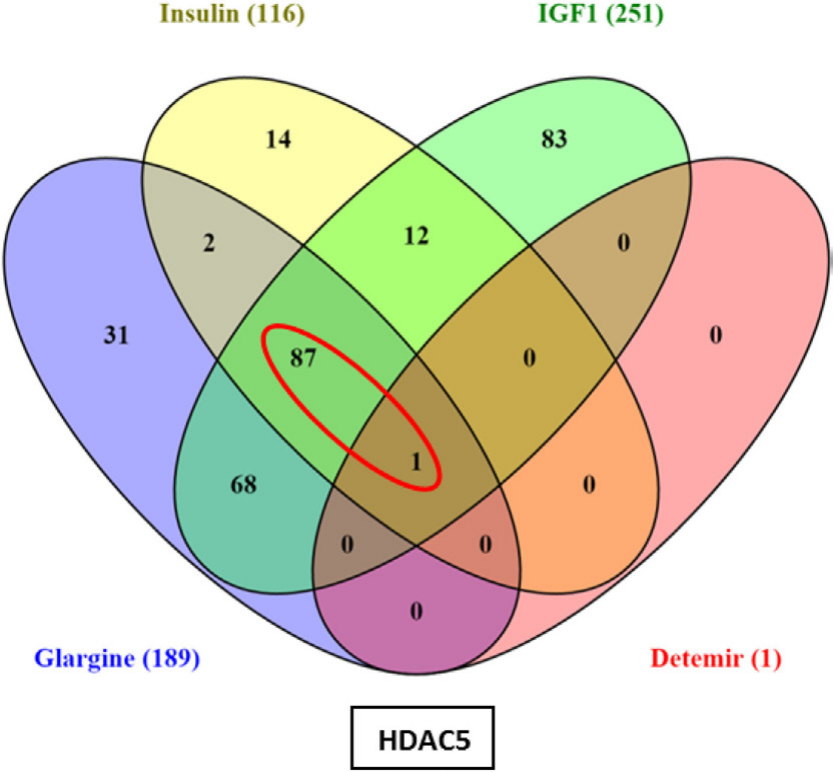
Venn diagram: differentially expressed genes in ECC-1 cells after ligands treatment. The diagram displays all possible logical relations between the differential gene expression sets for each treatment in ECC-1 cells. Only one gene (HDAC5) was differentially expressed after detemir treatment (shown in box). FC = 1.5 and pFDR < 0.05.

Hierarchical cluster analysis revealed differential expression of multiple genes in ECC-1 cells following stimulation with glargine (189 differentially expressed genes), IGF1 (251 differentially expressed genes), or insulin (116 differentially expressed genes), compared to untreated cells (Figures 1A–C). Principle component analysis (PCA) demonstrated that samples were nicely clustered (with a few exceptions) by treatment. There were variations between the different treatments, with detemir being the least effective treatment (Figure 1D). The Venn diagram shown in Figure 2 displays all possible logical relations between the differential gene expression sets for each treatment in ECC-1 cells. 298 genes were found to be differentially expressed by the four ligands in ECC-1 cells. In particular, IGF1-treated cells had the largest number of differentially expressed genes (251 genes), suggesting that it was the most potent treatment in comparison with the other ligands. Many of the differentially expressed genes upon treatment with insulin, glargine, or IGF1 are common to all three treatments (88 genes, circled in red). In contrast, detemir treatment was shown to be very similar to control cells gene expression. In fact, only one gene (histone deacetylase-5, HDAC5) was differentially expressed with stringent cutoff used (pFDR < 0.05 and FC = 1.5). Therefore, we chose to focus on the differentially expressed genes upon IGF1, glargine, or insulin treatments.

**Figure 3:**
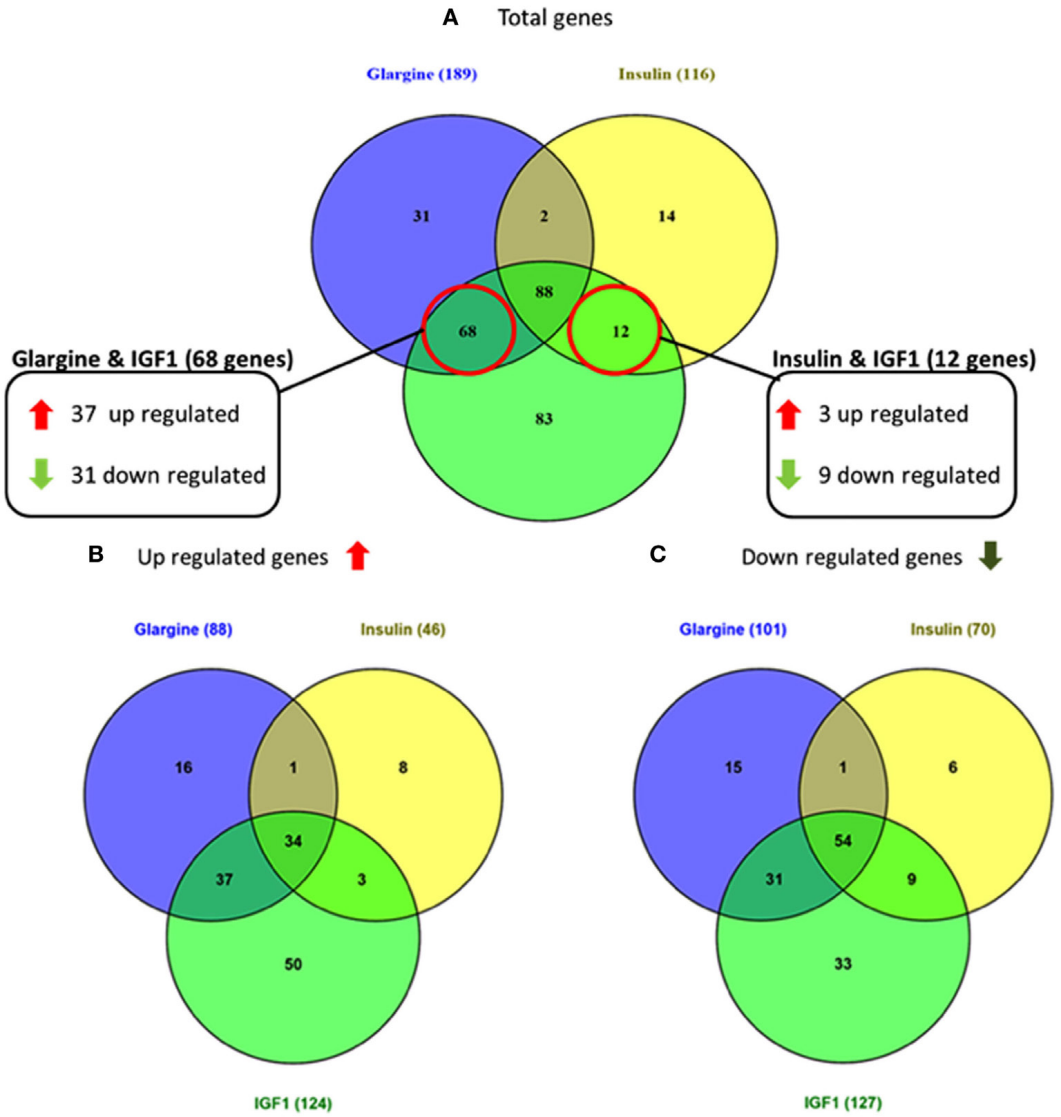
Venn diagrams showing the number of differentially expressed genes in response to glargine, regular insulin, or IGF1 in ECC-1 cells. **(A)** Number of total genes induced after IGF1, glargine, or regular insulin treatment; **(B)** number of upregulated genes; **(C)** number of downregulated genes. The Venn diagram shows the number of genes uniquely expressed after response to the indicated treatment. The intersection of the circles shows the number of genes commonly expressed (up- or downregulated). The genes included in this analysis showed a 1.5-fold change in expression compared to the control with a pFDR < 0.05.

As shown in Figure 3, 68 genes were differently regulated by both glargine and IGF1 treatments, while 12 genes were regulated differently by both insulin and IGF1. In percentages, 37% of the total genes induced by glargine were also induced by IGF1 treatment. On the other hand, only 10% of the genes induced by insulin were induced also by IGF1. Thus, treatment with glargine leads to the activation of more genes in common with IGF1 than with insulin.

**Figure 4:**
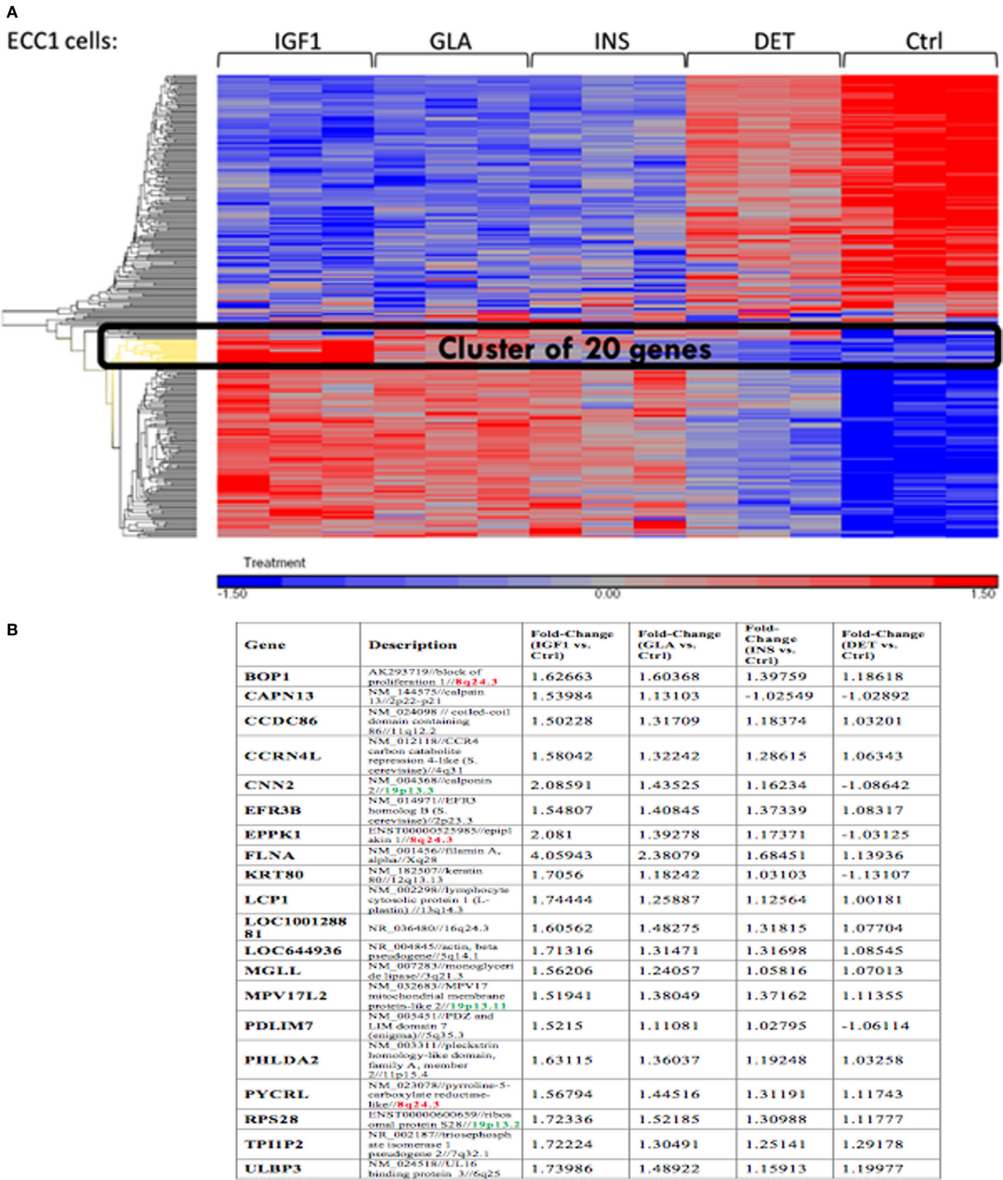
Features of differentially expressed genes in ECC-1 cells after ligands treatment. **(A)** Heatmap of 298 differentially expressed genes that were detected for all four treatments in ECC1 cells; **(B)** list of the 20 genes from the cluster marked in black in **(A)**. Fold changes are indicated.

Next, we conducted a pathway enrichment analysis of the differently expressed genes upon IGF1, insulin, or glargine treatments, compared to untreated ECC-1 cells. The enriched signaling pathway analysis revealed that, in addition to the mTOR, insulin, and IGF1 pathways, ligand stimulation alters additional pathways that are associated with cell cycle progression, proliferation, apoptosis, and migration, among others (Table 2). The features of gene expression changes in ECC-1 cells after treatment with all four ligands (glargine, detemir, IGF1, and insulin) are shown in Figure 4. Out of 298 differentially expressed genes detected with either one of the four treatments, clustering analysis identified the presence of a cluster of 20 genes. Except for detemir, these 20 genes showed the same differential expression in each treatment.

**Table 2:** Enriched signaling pathways following ligand stimulation in ECC-1 cells.

Pathway name	*p*-Value
mTOR signaling pathway	0.0017
ErbB1 downstream signaling	0.0017
GMCSF-mediated signaling events	0.0017
Signaling events mediated by VEGFR1 and VEGFR2	0.0017
EGFR-dependent endothelin signaling events	0.0017
PDGF receptor signaling network	0.0017
Endothelins	0.0017
Glypican 1 network	0.0017
Plasma membrane estrogen receptor signaling	0.0017
Class I PI3K signaling events mediated by Akt	0.0017
VEGF and VEGFR signaling network	0.0017
IGF1 pathway	0.0017
IRS activation	0.0017
Signaling events mediated by focal adhesion kinase	0.0017
PDGFR-beta signaling pathway	0.0017
Insulin pathway	0.0017
Alpha9 beta1 integrin signaling events	0.0017
Proteoglycan syndecan-mediated signaling events	0.0017
Class I PI3K signaling events	0.0017
LKB1 signaling events	0.0017
Signaling events mediated by hepatocyte growth factor receptor (c-Met)	0.0017
IFN-gamma pathway	0.0017
S1P1 pathway	0.0017
Sphingosine 1-phosphate pathway	0.0017
ErbB receptor signaling network	0.0017
Beta1 integrin cell surface interactions	0.0023
Integrin family cell surface interactions	0.0029
Signaling events mediated by HDAC Class II	0.0063
G1/S-specific transcription	0.0145
Growth hormone receptor signaling	0.0179
G0 and Early G1	0.0238
Signaling events mediated by PRL	0.0271
Signaling events regulated by Ret tyrosine kinase	0.029
E2F transcription factor network	0.0329
G2/M DNA damage checkpoint	0.0366
Mitotic G1-G1/S phases	0.0366
Androgen receptor	0.0366
Insulin receptor recycling	0.0366

**Figure 5:**
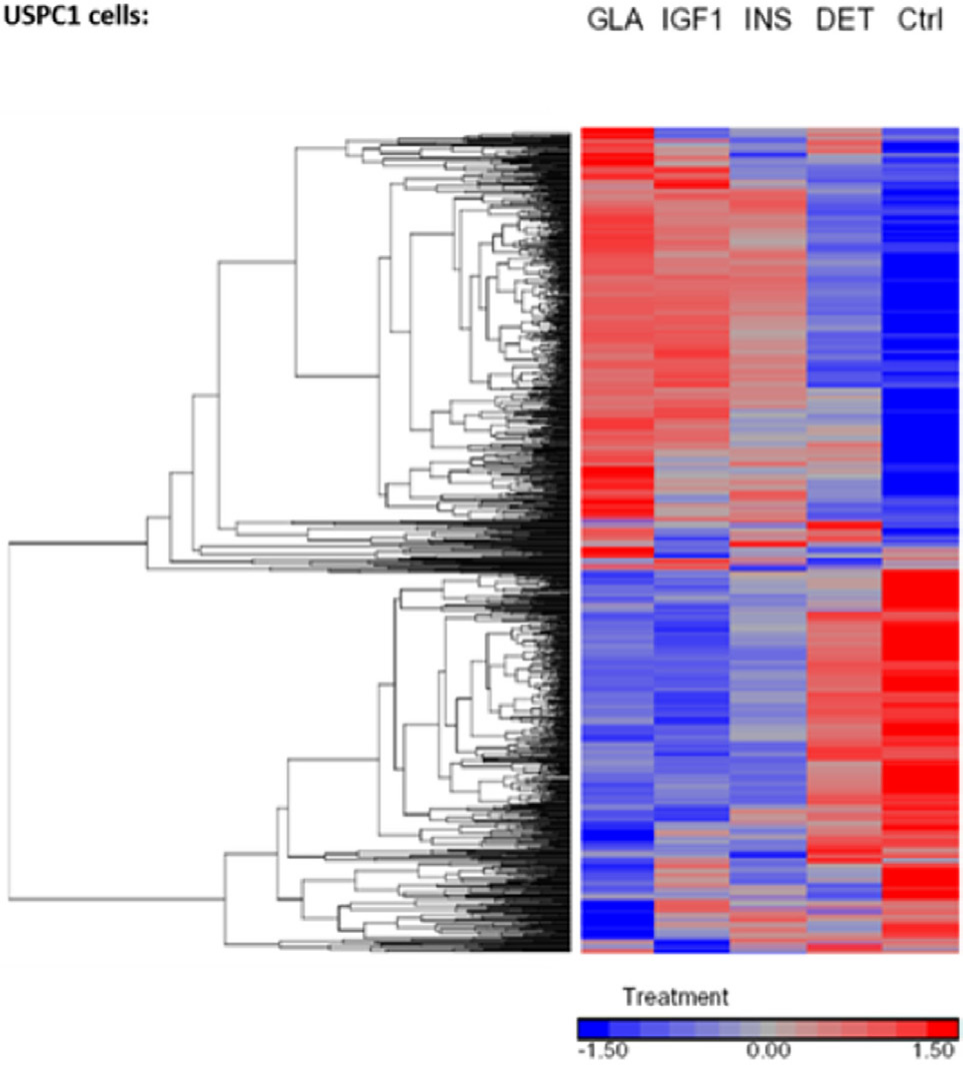
Hierarchical clustering map of differential gene expression in USPC-1 cells. Heatmaps of microarray results in the USPC-1 cell line after treatment with insulin glargine, detemir, IGF1, and regular insulin. The figure depicts a heatmap of differentially expressed genes (FC > 1.5 or <−1.5). The scale below indicates the relative changes in gene expression represented by red and blue colors. Upregulated genes are shown in red and downregulated genes are shown in blue. FC, fold change.

**Figure 6:**
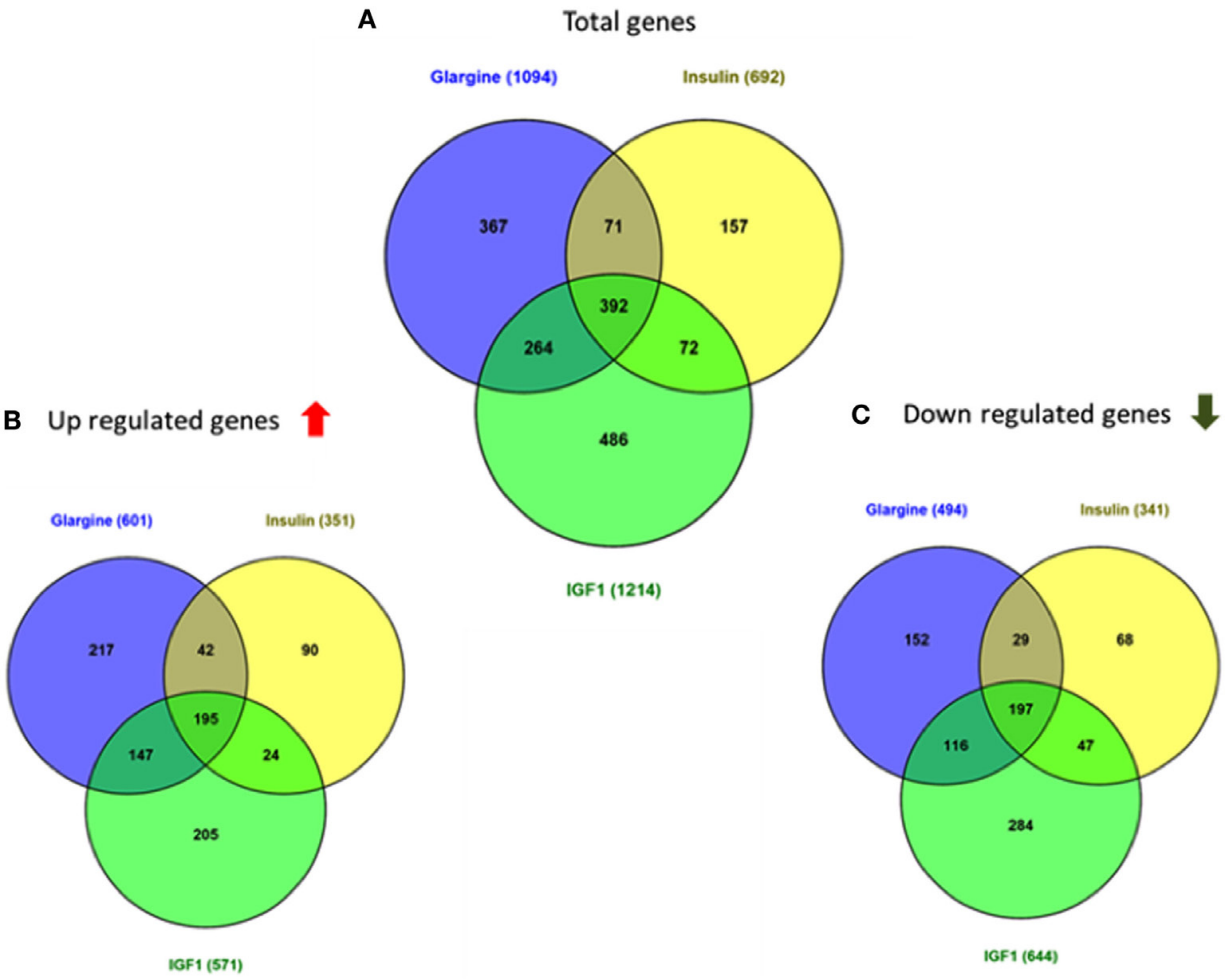
Venn diagrams showing the number of differentially expressed genes in response to glargine, insulin, or IGF1 in USPC-1 cells. **(A)** Number of total genes induced after IGF1, glargine, or insulin treatment; **(B)** number of genes up-regulated; **(C)** number of genes downregulated. The Venn diagram shows the number of genes uniquely expressed after response of the indicated treatment. The intersection of the circles shows the number of genes commonly expressed. The genes included in this analysis showed a 1.5-fold change in the expression compared to the control.

**Figure 7:**
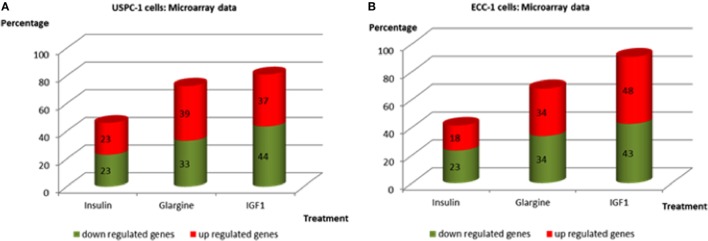
Commonly up- and downregulated genes by regular insulin, glargine, and IGF1 treatments in USPC-1 cells **(A)** and in ECC-1 cells **(B)**. Comparison of common genes up- and downregulated by insulin, glargine, and IGF1 (in percentages) in both cell lines.

Regarding USPC-1 cells, microarray analysis demonstrated a similar expression pattern to ECC-1 cells. The hierarchical clustering map shows that detemir had very little effect in comparison to the other treatments (Figure 5). Likewise, the other three treatments (glargine, IGF1, and insulin) exhibited similar effects with minor differences. Cluster analysis detected 3,000 differentially expressed genes for the four treatments in these cells (fold-change difference = 1.5). Similarly to ECC-1, we investigated the total, up- and downregulated expression of genes elicited by the hormones (Figure 6). Like ECC-1, treatment with glargine had more genes in common with IGF1 treatment than with insulin (Figure 6A). Furthermore, Venn analysis showed a similar number of up- and downregulated common genes induced by all three ligands (Figures 6B,C). Moreover, ECC-1 and USPC-1 cells showed the same percentage of up- and downregulated commons genes after treatment by all three ligands (Figure 7). The difference in number of genes compared to ECC-1 cells could be explained by the fact that the microarray analysis of USPC-1 cells was performed only in five samples (corresponding to one biological replicate per treatment). Therefore, clusters analysis was done on the basis of FC = 1.5 only, without a pFDR statistical value available.

### Microarray Data Validation

**Figure 8:**
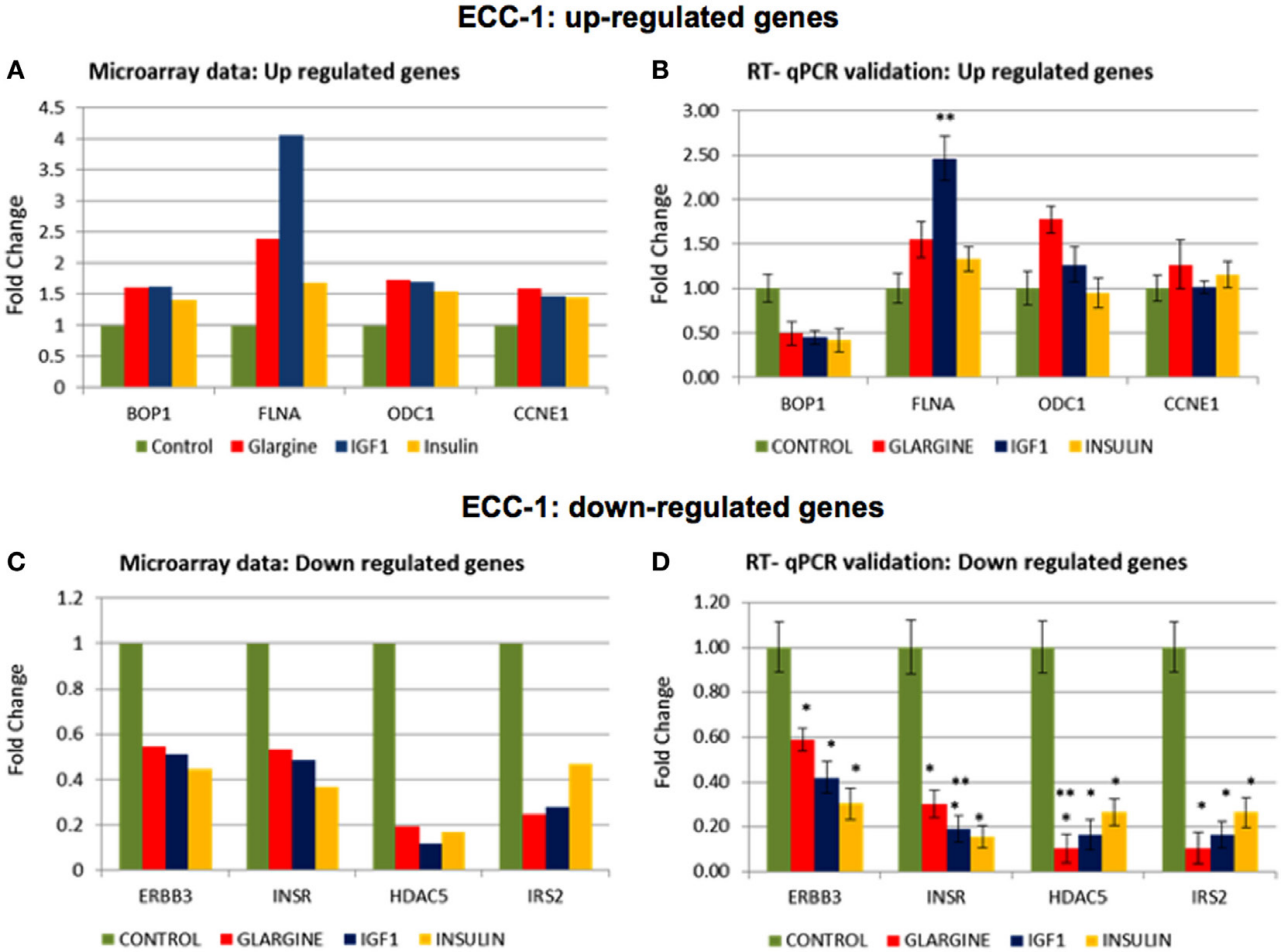
Real-time quantitative polymerase chain reactions (RT-qPCR) validation of the microarray data for selected up- and downregulated genes in ECC-1 cells. **(A)** Microarray results of four selected upregulated genes; **(B)** relative expression of the upregulated genes by RT-qPCR. **(C)** Microarray results of four selected downregulated genes; **(D)** relative expression of the downregulated genes by RT-qPCR. Lower fold-change values were obtained in the RT-qPCR validation compared to microarray assays. RT-qPCR was done in triplicate and the ratio was calculated relative to the reference gene actin. Statistical analysis was done by using Student’s test. *p < 0.05 insulin analogs vs. untreated cells; **p < 0.05 ligand vs. regular insulin.

**Figure 9:**
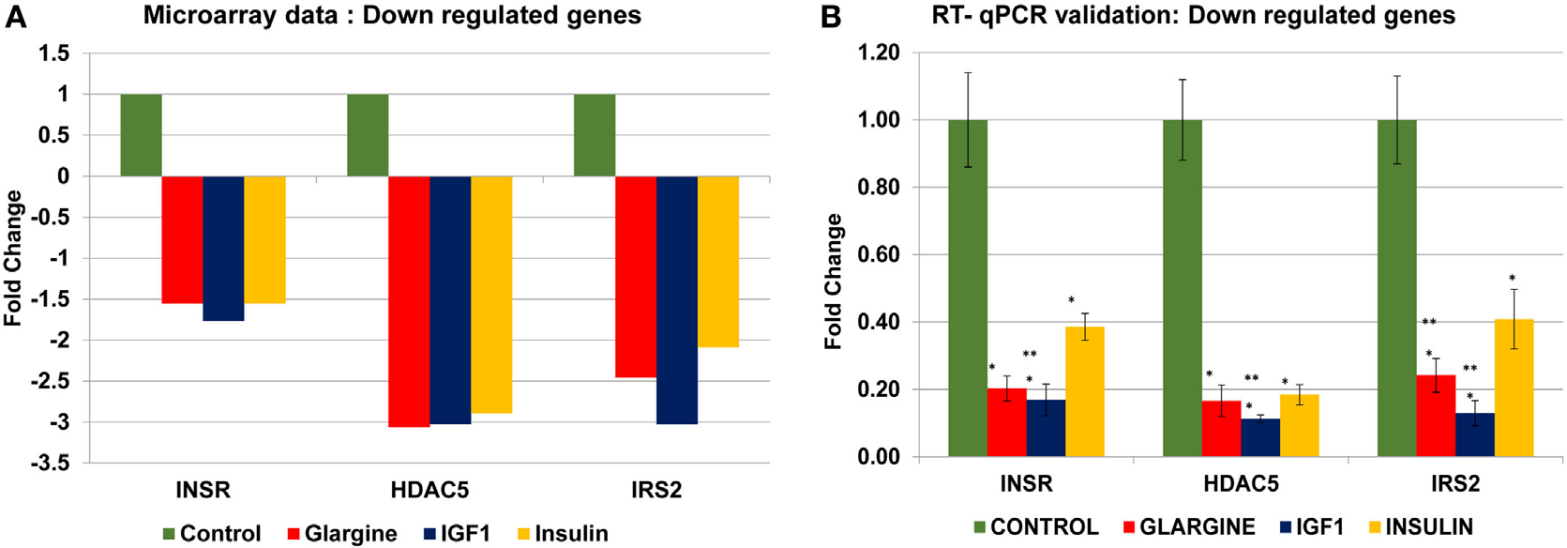
Real-time quantitative polymerase chain reactions (RT-qPCR) validation of the microarray data for selected downregulated genes in USPC-1 cells. **(A)** Microarray results of four selected downregulated genes; **(B)** relative expression of the downregulated genes by RT-qPCR. Lower values were obtained in the RT-qPCR validation compared to values obtained in the microarray assay. RT-qPCR was done in triplicate and the ratio was calculated relative to actin. *p < 0.05 insulin analogs vs. untreated cells; **p < 0.05 ligand vs. regular insulin. The *y*-axis represents fold changes.

In order to validate the microarray results in ECC-1 and USPC-1 cells, changes in mRNA levels of four upregulated genes [cyclin E1 (CCNE1), FLNA, ornithine decarboxylase (ODC1), block of proliferation] and four downregulated genes [Erb-B2 receptor tyrosine kinase (ERBB3), insulin receptor (INSR), histone deacetylase-5 (HDAC5), insulin receptor substrate-2 (IRS2)], were quantified using RT-qPCR from new independent RNA samples after ligand treatment (8.6 nM, 8 h). These genes were associated with cell cycle, cellular assembly and organization, cell proliferation, cellular growth and development, and DNA synthesis and repair. RT-qPCR results confirmed the microarray data for two of the upregulated genes (FLNA, ODC1) (Figures 8A,B) and for the four downregulated genes (ERBB3, INSR, HDAC5, IRS2) (Figures 8C,D). RT-qPCR results of the BOP and CCNE1 genes did not support the microarray gene expression data. In contrast, we did not find correlation between microarray gene expression data and RT-qPCR values for upregulated genes in USPC-1 cells (data not shown). RT-qPCR results of downregulated genes in USPC-1 cells (HDAC5, INSR, and IRS2) confirmed the microarray data (Figure 9). The relatively poor validity may be explained by the fact that the USPC-1 experiment was performed on single replicates only.

### Identification of FLNA As a Target for Insulin and IGF1 Action

**Figure 10:**
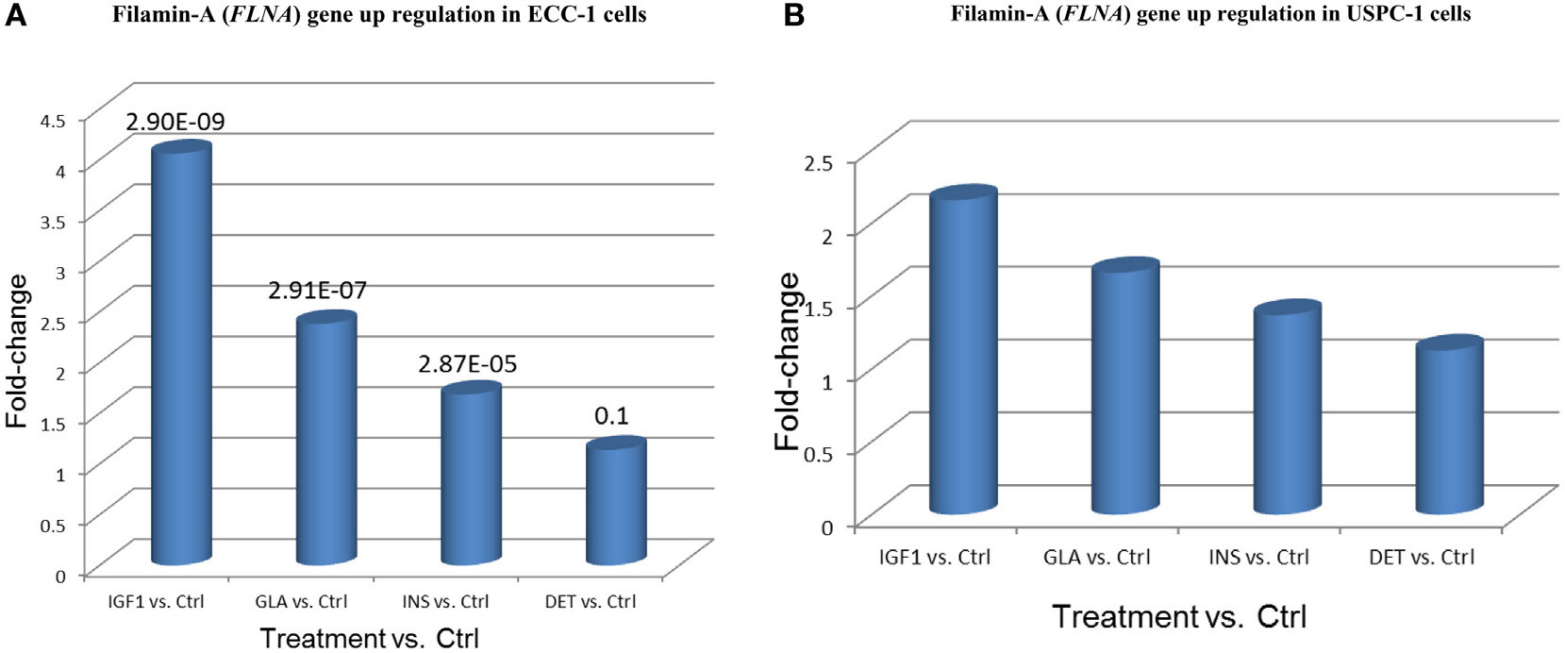
Filamin-A (*FLNA*) gene upregulation in **(A)** ECC-1 and **(B)** USPC-1 cells. Starved ECC-1 **(A)** and USPC-1 **(B)** cells were treated with IGF1, glargine, insulin, or detemir at a dose of 8.6 nM for 24 h, after which, RNA was prepared and *FLNA* gene expression was measured as described in Section “Materials and Methods.” Results are expressed as fold-changes compared to untreated cells.

The *FLNA* gene encodes an actin-binding protein involved in the cross-linking of actin filaments and membrane glycoproteins. The FLNA protein has an important role in remodeling the cytoskeleton with ensuing changes in cell shape and migration. In addition, FLNA interacts with transmembrane receptor complexes, integrins, and second messengers. As indicated above, the *FLNA* gene was identified as member of a cluster of 20 genes that exhibited the same differential expression following each treatment. Furthermore, among all validated candidate genes, *FLNA* displays the greatest fold-changes compared to untreated cells (4.05-FC for IGF1 treatment, 2.38 for glargine, and 1.68 for insulin) (Figure 10).

**Figure 11:**
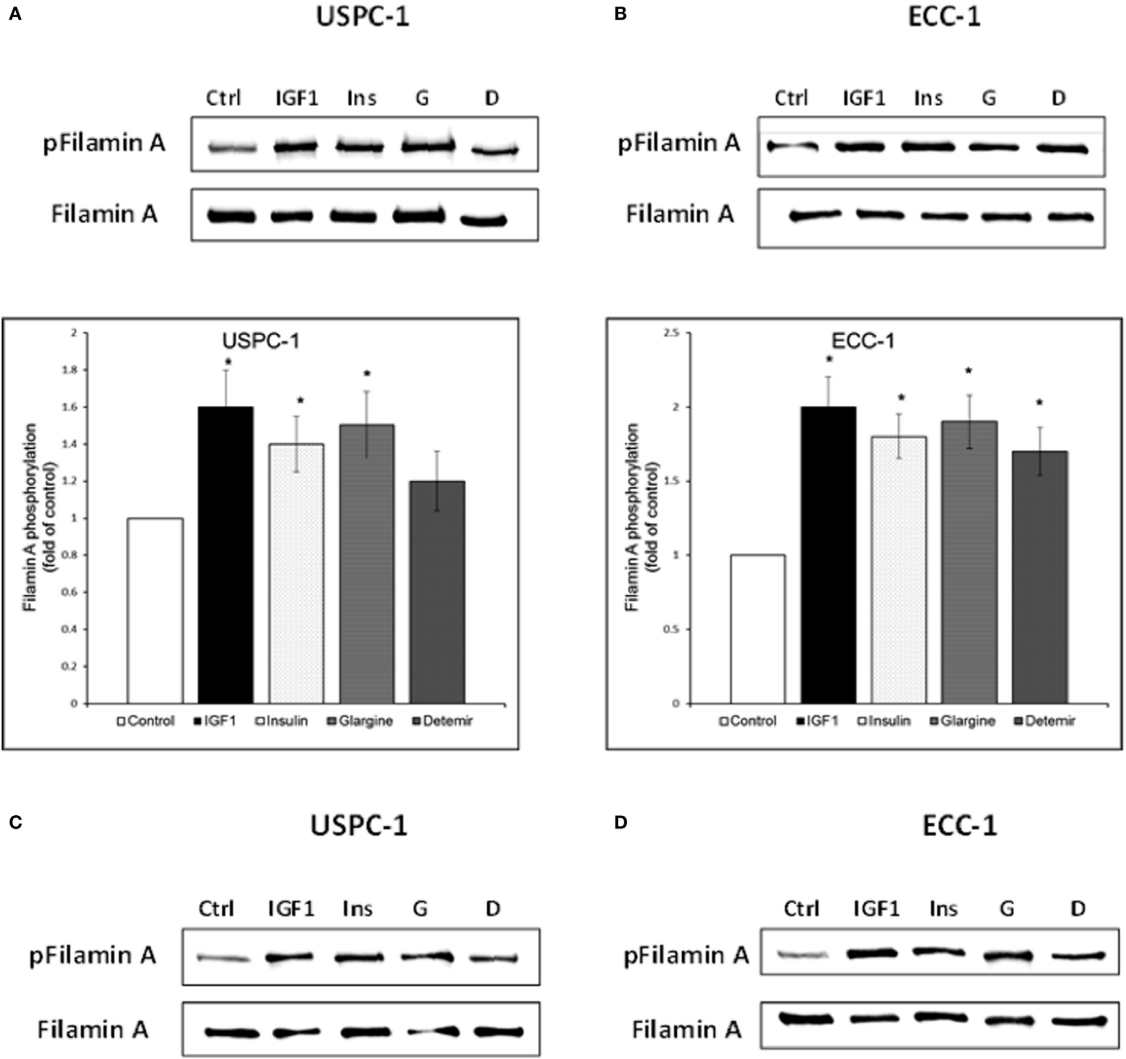
Filamin-A (FLNA) phosphorylation by insulin-like hormones. Starved USPC-1 and ECC-1 cells were treated with IGF1, insulin, glargine, or detemir at a dose of 50 ng/ml for 10 min **(A,B)**, or 8 h **(C,D)**. Cells were lysed and extracts (100 µg) were electrophoresed and immunoblotted with antibodies against phospho-filamin-A. Membranes were stripped and incubated with antibodies against total FLNA. Fold of control was obtained by normalizing levels of the phospho-protein by total protein levels and then comparing with untreated cells. Bar graphs represent three independent experiments; *p < 0.05, ligand-treated vs. untreated.

To validate the reliability of the microarray and RT-qPCR data regarding FLNA, we next analyzed FLNA protein expression by Western immunoblots. To this end, USPC-1 and ECC-1 cells were treated with glargine, detemir, IGF1, or insulin for 10 min or 8 h, after which, levels of total and phosphorylated FLNA were measured. As depicted in Figures 11A,B, treatment with all four ligands led to substantial increases in phosphorylated FLNA levels after 10 min, with a similar pattern in both cell lines. Interestingly, IGF1 induced the highest increase in FLNA phosphorylation (1.6-fold in USPC-1 cells and 2-fold in ECC-1 cells). Treatment with glargine or insulin induced similar levels of FLNA phosphorylation (approximately 1.5-fold increase in USPC-1 cells and 1.9-fold increase in ECC-1 cells) in both cell lines. Of interest, detemir stimulated FLNA phosphorylation in ECC-1 cells similarly to the other hormones. Increases in phosphorylation were also seen at 8 h (Figures 11C,D).

### Silencing Experiments

**Figure 12:**
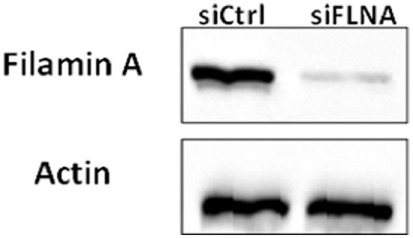
Western blot analysis of FLNA expression in FLNA-silenced cells. ECC-1 cells were transfected with 10 µM control siRNA (siCtrl) or FLNA siRNA (siFLNA) for 96 h. Cells were lysed and extracts (100 µg) were electrophoresed and immunoblotted with an antibody against FLNA. Actin levels were measured as a loading control. The figure shows the results of a typical experiment, repeated three times with similar results.

**Figure 13:**
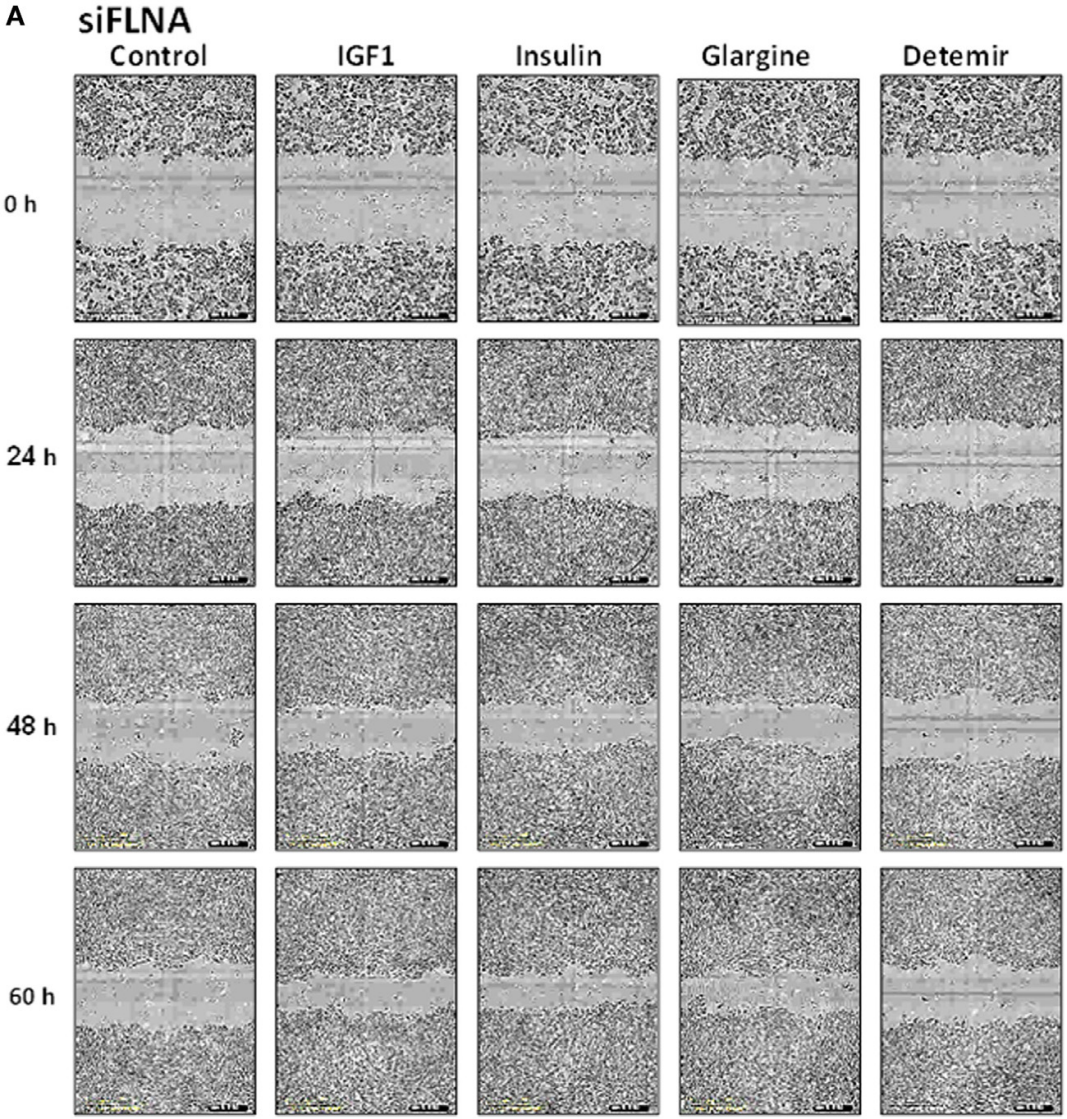

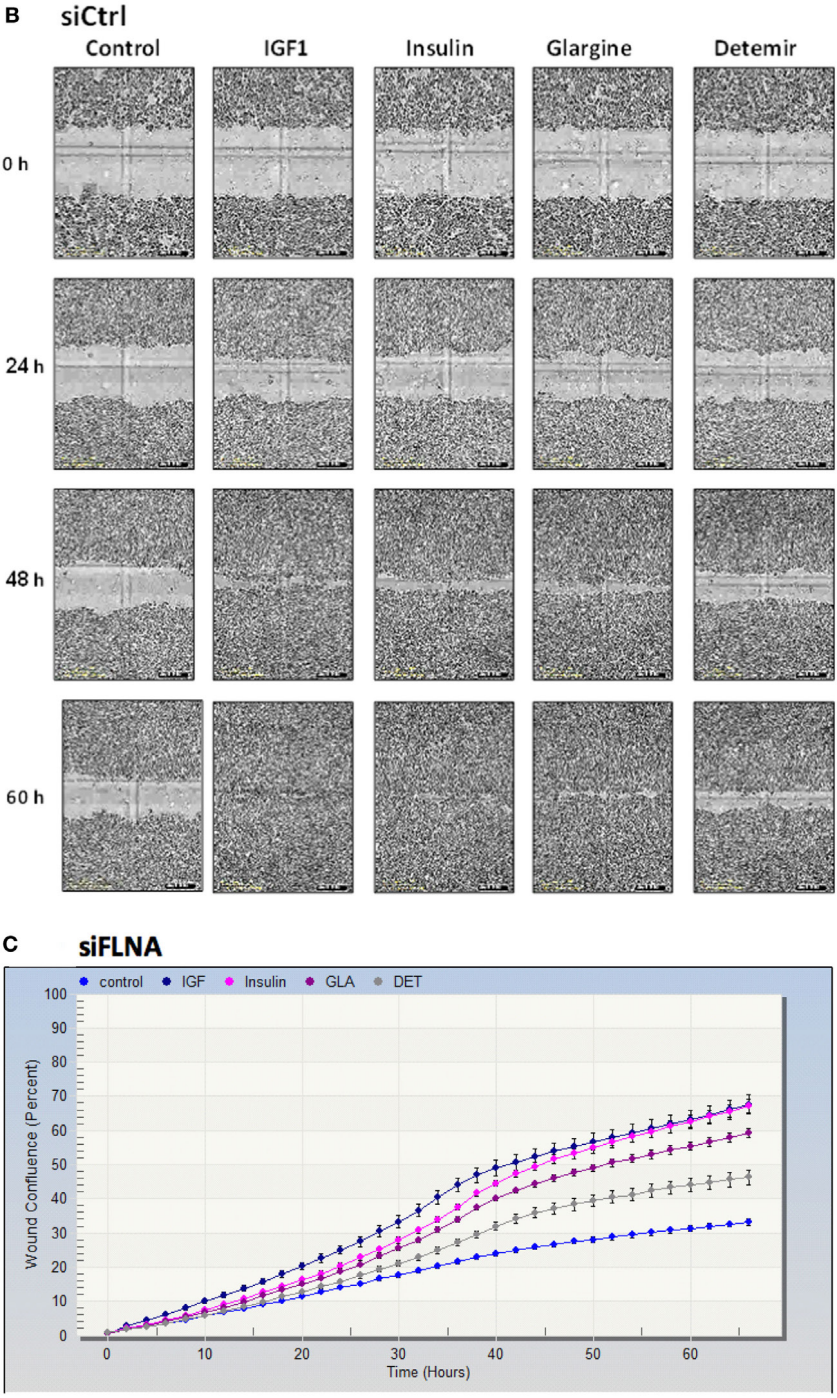

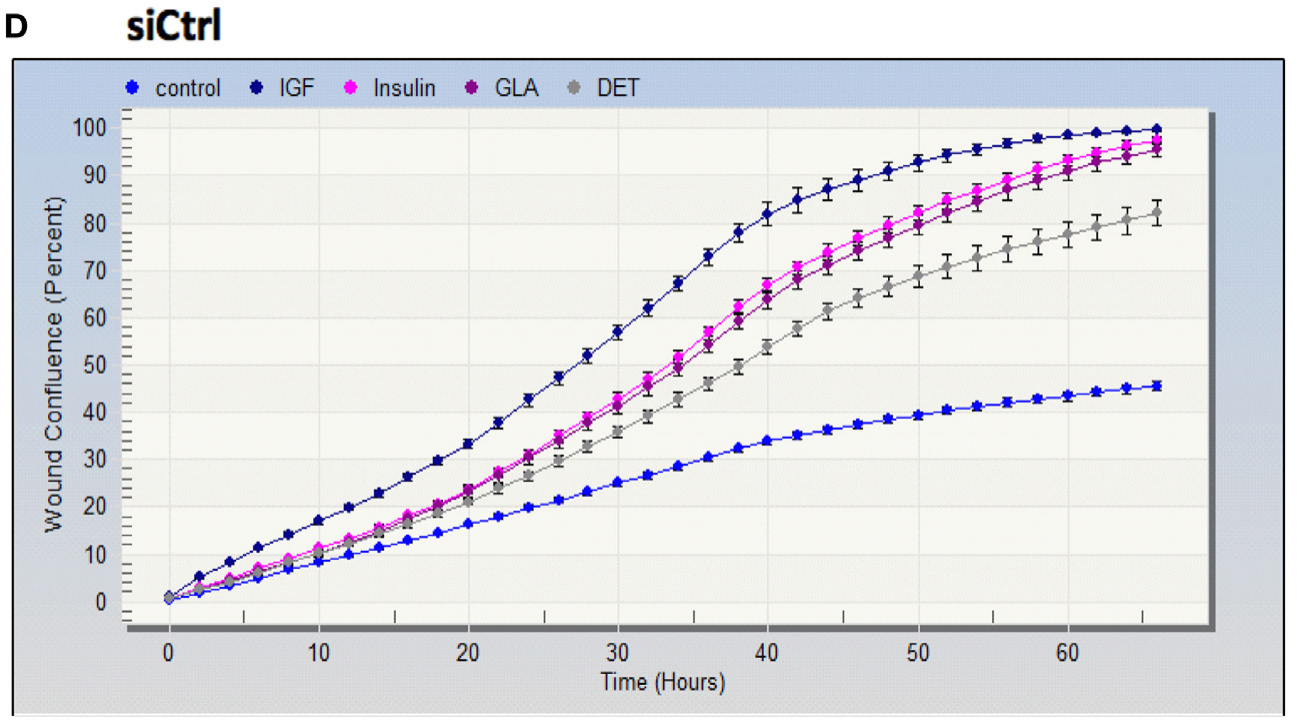
Cell migration measurement in FLNA-silenced cells. Cell migration assays were performed in ECC-1 cells transfected with 10 µM siFLNA (or siCtrl), as described in Section “Materials and Methods.” Cells were scratch wounded and monitored to determine the rate of migration into the scratched area. Representative images indicate areas of the scratch wound that are occupied by ECC-1 cells transfected with siFLNA **(A)** or siCtrl **(B)**, at 0 (initial scratch), 24, 48, and 60 h post-wounding. Results were plotted in terms of percentage wound confluence as a function of time **(C,D)**. Data are expressed as mean ± SD, *n* = 10.

Evidence has been presented showing that FLNA has an important role in mammalian cell locomotion (17). The potential hormonal regulation of this process has not yet been investigated. Given that IGF1, insulin, and analogs were shown to phosphorylate FLNA, we postulated that hormonal activation of FLNA might have a key role in cell migration. To evaluate this hypothesis, we silenced FLNA by transfecting ECC-1 cells with a FLNA-specific siRNA (siFLNA), or control scramble sequence (siCtrl), followed by ligand (17.2 nM) treatment for 60 h. Western blots indicated that siFLNA transfection markedly reduced FLNA protein levels (Figure 12). Cell migration measurements showed that ligand treatment increased ECC-1 cells migration compared to controls. A marked reduction in migration was observed in siFLNA-transfected, compared to siCtrl, cells. Thus, total wound closure was seen after 60 h in control cells treated with insulin, IGF1, or glargine (Figures 13B,D). On the other hand, a strong inhibition of ligand-induced migration was observed in cells with a silenced FLNA (Figures 13A,C). These results indicate that (1) FLNA is an important regulator of cell migration; and (2) all four ligands tested stimulated FLNA activation.

## Discussion

The introduction of insulin analogs in the last decades had a great impact in the area of diabetes. The higher stability, reduced variability and, importantly, selective action of insulin analogs led to an increase in individualized treatment strategies targeted to specific patient needs. In general terms, advances resulted in improvement in glycemic control and diabetes therapy (18–20). However, modifications in the insulin molecule may change the affinities of the analogs for INSR and IGF1R as well as their intracellular signaling and biological effects (21). Hence, the long-term benefit of some of the new insulin analogs has been questionated.

In recent years, the medical literature has become aware that the use of some long-acting analogs was correlated with an apparent increase in cancer risk. Specifically, large-scale epidemiological studies published in 2009 raised the concern that insulin analogs, especially glargine, might increase breast cancer risk (22–26). Some of these analyses, however, have been criticized on the basis of study design (27, 28). These controversies emphasize the need for more clinical and basic research to clarify this issue. In view of flaws in study design and analyses of these “first generation” studies, “second generation” studies emerged with more robust pharmaco-epidemiological analyses (29). Out of five “second generation” studies, only one observed a significantly increased breast cancer risk associated with high cumulative exposure to glargine (30–34). However, all studies lacked sufficient follow-up to robustly estimate cancer risk. Until now, there is no epidemiological and animal evidence that any clinically available insulin analog, nor human insulin, increases breast cancer risk. In any case, several studies have suggested that although insulin treatment was not involved in breast tumor initiation, it might induce tumor progression by upregulating mitogenic pathways.

In the present study, microarray analysis revealed alterations in genes and cancer-associated pathways following ligand stimulation. Indeed, hierarchical cluster analysis identified 298 and 3,000 differentially expressed genes in ECC-1 and USPC-1 cells lines, respectively. Our analyses revealed that IGF1, insulin, and glargine stimulate overlapping genes (88 genes in ECC-1 cells and 392 in USPC-1 cells), as well as specific gene expression responses. Furthermore, we showed that most signaling pathways activated by the ligands are involved in cell cycle progression, proliferation, apoptosis, and cell migration. Of interest, we provide evidence that each ligand specifically regulated a group of genes that was not regulated by the other two ligands. For example, in ECC-1 cells, we observed that 83 genes were significantly induced by IGF1 whereas 31 genes and 14 genes were specifically regulated by glargine and insulin, respectively. These results demonstrate that glargine activates distinct sets of genes that are associated to specific pathways that are not necessarily activated by IGF1 or insulin. Moreover, we showed the same percentage of up- and downregulated commons genes in ECC-1 and USPC-1 cells lines after treatment with insulin, glargine, or IGF1.

For the most part, RT-qPCR validation experiments confirmed that the microarray data identified changes in gene expression mediated by these ligands. Validated genes are involved in various cellular functions, including cytoskeleton remodeling and migration (FLNA), biosynthetic pathways (ODC1), cell proliferation (ERBB3), cytoplasmic insulin signaling (INSR, IRS2), and cell cycle progression and developmental events (HDAC5).

Global gene expression changes following IGF1 or insulin treatment have been examined in multiple mouse and human cell lines (35–38). Dupont et al. (35) evaluated the IGF1-induced global gene expression in mouse NIH-3T3 fibroblasts overexpressing either IGF1R or INSR in an attempt to understand how insulin and IGF1 can signal through the same post-receptor signaling pathways, yet mediate distinct biological functions. They found that 30 out of 2,221 genes were significantly induced by IGF1 but not by regular insulin; most of these genes were associated with mitogenesis and differentiation. Similarly to our study, Dupont et al. found that IGF1 appeared to have a greater stimulatory effect on transcription regulation than insulin.

Our microarray analysis identified FLNA as one of the most significantly upregulated genes induced by glargine, IGF1, or insulin in the ECC-1 cell line. FLNA was initially identified as an actin filament cross-linking protein that also interacts with integrins and a number of cytoskeleton remodeling proteins (39). FLNA’s importance in cellular adhesion and migration makes it a critical player in cancer invasion and metastasis (40, 41). Moreover, FLNA functions as a scaffold for about 90 binding partners and is involved in many cellular functions, including cell signaling, motility, angiogenesis, phosphorylation, proteolysis, transcription, receptor activation, muscle development, etc. (42–44). Of importance, FLNA anchors the GTPases to the cell membrane. Western blots revealed that FLNA is basally expressed in both ECC-1 and USPC-1 endometrial cancer cells. This finding is in accordance with Brown and Binder (45), who reported high filamin expression in uterus, among other organs. Furthermore, our results are in line with the fact that FLNA is overexpressed in multiple types of cancer (41).

The biological actions of insulin are associated with a rapid and efficient reorganization of the cytoskeleton. The specific effect of FLNA on insulin-mediated signaling events is still unknown. Further, limited information is available regarding the question whether insulin analogs are also capable of regulating the key processes of tumor cell motility, invasion, and metastasis. We and others have reported that glargine is a potent activator of protein kinase Akt in endometrial cancer cell lines (16). Evidence has suggested that Akt can not only provide the cells with growth advantages by eliciting proliferative or antiapoptotic signals but also engage with motility of the cells through regulating actin stress fiber assembly (46). Thus, it is possible that glargine participates in tumor cell migration through Akt activation and subsequent FLNA phosphorylation.

In summary, we have identified FLNA as a novel target for insulin-like hormones, including glargine. Further studies will dissect the physical and functional interactions between insulin-like hormones and FLNA, and the impact of these interactions on endometrial cancer motility, invasion, and metastasis. Likewise, it will be of interest to examine the biological roles played by the other genes identified by genomic tools in the specific context of the IGF1R and INSR pathways.

## Author Contributions

DA, IB, and HW conceived of and designed the experiments. The experimental procedures were performed by DA and RS and were analyzed by DA, MP-C, and HW. HW and ZL prepared the manuscript.

## Conflict of Interest Statement

The authors declare that there is no conflict of interest that could be perceived as prejudicing the impartiality of the research reported.
